# Author Correction: Targeted memory reactivation in REM but not SWS selectively reduces arousal responses

**DOI:** 10.1038/s42003-021-02196-w

**Published:** 2021-05-21

**Authors:** Isabel C. Hutchison, Stefania Pezzoli, Maria-Efstratia Tsimpanouli, Mahmoud E. A. Abdellahi, Gorana Pobric, Johann Hulleman, Penelope A. Lewis

**Affiliations:** 1 Department of Neurology, Northwestern University, Chicago, IL USA; 2 Department of Neuroscience, University of Sheffield, Sheffield, UK; 3 Department of Neurology and Sleep Disorders Center, University of Michigan, Ann Arbor, MI USA; 4 School of Psychology, Cardiff University, Cardiff, UK; 5 Division of Neuroscience & Experimental Psychology, University of Manchester, Manchester, UK; 6 Present Address: Molecular Biophysics and Integrated Bioimaging, Lawrence Berkeley National Laboratory, Berkeley, CA USA; 7 Present Address: Helen Wills Neuroscience Institute, University of California Berkeley, Berkeley, CA USA

**Keywords:** Human behaviour, Circadian rhythms and sleep, Cognitive neuroscience, Emotion, Learning and memory

Correction to: *Communications Biology* 10.1038/s42003-021-01854-3, published online 25 March 2021.

The original version of the Article inadvertently omitted the following authors from the author list:

- Johan Hulleman, Division of Neuroscience & Experimental Psychology, University of Manchester, Manchester, UK.
- Gorana Pobric, Division of Neuroscience & Experimental Psychology, University of Manchester, Manchester, UK.

The new author list is as follows: Isabel C. Hutchison, Stefania Pezzoli, Maria-Efstratia Tsimpanouli, Mahmoud E. A. Abdellahi, Gorana Pobric, Johann Hulleman & Penelope A. Lewis.

The Author contributions section has been amended to include the following:

- G.P.—assisted in design of the task, analysis and interpretation
- J.H.—assisted in analysis and interpretation

The acknowledgements section has also been amended to include the text shown here in bold font: BBSRC (BB/J014478/1) DTP studentship **awarded to I.C.H., P.A.L. and G.P**.

Additionally, the affiliations for author Stefania Pezzoli have been updated to include two Present Address affiliations:

1. Molecular Biophysics and Integrated Bioimaging, Lawrence Berkeley National Laboratory, Berkeley, CA, USA
2. Helen Wills Neuroscience Institute, University of California Berkeley, Berkeley, CA, USA

All changes have been made in the HTML and PDF versions of the Article.

