# Supplementary material for: Targeted memory reactivation in REM but not SWS selectively reduces arousal responses
Source: Commun Biol. 2021 Mar 25;4:404. doi: 10.1038/s42003-021-01854-3 (PMC7994443; doi:10.1038/s42003-021-01854-3)
Supplement: Supplementary file 2 — Supplementary Information [file 42003_2021_1854_MOESM2_ESM.pdf]

Targeted memory reactivation in REM but not SWS selectively reduces arousal responses

Isabel C. Hutchison, Ph.D.[1]

Stefania Pezzoli, Ph.D.[2]

Maria-Efstratia Tsimpanouli, Ph.D.[3]

Mahmoud E. A. Abdellahi[5]

Penelope A. Lewis, Ph.D.[4,5] *

*corresponding author: lewisp8@cardiff.ac.uk

[1]Department of Neurology, Northwestern University, 303 East Chicago Avenue, 60611 Chicago, IL, USA
[2]Department of Neuroscience, University of Sheffield, 385a Glossop Road, S10 2HQ Sheffield, UK,
[3]Department of Neurology and Sleep Disorders Center, University of Michigan, 1500 E. Medical Center Drive, 48109 Ann Arbor, MI, USA
[4]Division of Neuroscience & Experimental Psychology, University of Manchester, Oxford Road, M13 9PL, Manchester, UK
[5]School of Psychology, Cardiff University, Cardiff, UK

Supplementary Material

Supplementary Table S1: Reaction times and standard deviations from pre-sleep, post-sleep, and change between these. Outliers >= three standard deviations were removed from the analysis, resulting in removal of one participant from the neutral non-cued category in the REM group, and 1 participant both neutral and negative non-cued categories in the SWS group. Inclusion of these outliers did not change the results.

			Pre-Sleep Exposure	Post-Sleep Exposure	Change (Post-Pre)
**REM Group**	**Neutral**	**Cued**	936.9 ± 412.4	809.4 ± 195.03	-127.5 ± 157.9
		**Un-Cued**	984.1 ± 254.2	766.3 ± 273.7	-217.9 ± 259.0
	**Negative**	**Cued**	1040.7 ± 342.7	822.3 ± 245.3	-218.4 ± 284.0
		**Un-Cued**	966.6 ± 160.5	839.0 ± 238.2	-125.7 ± 194.5
**SWS Group**	**Neutral**	**Cued**	767.8 ± 147.6	641.2 ± 148.0	-126.5 ± 92.5
		**Un-Cued**	805.0 ± 182.3	669.3 ± 110.4	-149.2 ± 109.0
	**Negative**	**Cued**	906.2 ± 197.7	695.7 ± 152.6	-210.6 ± 93.2
		**Un-Cued**	943.8 ± 177.7	782.3 ± 175.4	-151.3 ± 192.0

Supplementary Table S2 Neutral and negative IAPS images

30 neutral and 30 negative images from the International Affective Picture System (IAPS) were selected for this study. Pictures were converted to grey scale and matched in luminance and resolution to minimize light-specific variation in respect to pupil dilation. A description of the content of each of the stimuli is listed separately for negative and neutral images.

Negative IAPS	Neutral IAPS
Pitbull	Tropical bird
Shark	Farmer
Toddler with flies on face	Neutral male face
Grieving female with corpse	Female secretary on phone
Crying child	Couple walking down stairs
Car accident corpse	Sleeping males on train
Male corpse on train	Two females chatting
Battered female	Female binge eating
Crying male in hospital	Female tourist
Unconscious male on ventilator	Male blow-drying hair
Disabled child	Towel
Bandaged child	Spoon
Distressed female	Porcelain bowl
Military ground attack	Mug
Female toddler crying at male	Fan
Roach on Pizza	Beer glass
Cemetery	Glass candle stick
Rotting cow carcass	Lightbulb
Power plant	Dice
Toilet soiled with feces	Fork
Toilet filled with vomit	Book

Sliced Hand	Truck
Crying soldier	Bus
Male threatened at gun point	Bedside lamp
Crime scene with body	Carpet
Human skulls	Clothes rack
Dead soaked cat	Plate with duck pattern
Burning jet	House
Filled ashtray	Traffic jam
Car Accident	Box of tissues
